# Results of Different Modes of Treatment of Cholera

**Published:** 1856-10

**Authors:** 


### *Results of different modes of Treatment of Cholera.*

The General Board of Health of England, which labored with so much zeal to stay the cholera epidemic of 1854, by the establishment of appropriate hygienic precautions, and by acquainting the public with the efficacy of medical interference in the premonitory stage of the malady, has lately published, as a fit conclusion of its duties, a statistical report of 3,727 cases of cholera, with the results of the different methods pursued in their treatment. Documents emanating from such a respectable source are certainly entitled to our notice, however wary we may be in lending confidence to cholera statistics in general.

The cases are arranged in four classes, according to the kind of medication to which they were subjected. Distinctions are also established, having reference to the period at which the cases were put under treatment. We must content ourselves with recapitulating the number of cases, the nature of the remedy, and the ratio of mortality.

Class I.—*Treated by Alteratives*, 2,142 *Cases, as follows:*

| Treated by | Cases. | Deaths. | Per centage. |
|---|---|---|---|
| Calomel, small doses, | 637 | 315 | 49.4 |
| Calomel, large doses, | 767 | 353 | 46. |
| Calomel, with opium, | 472 | 169 | 35.8 |
| Other mercurials, | 80 | 42 | 52.5 |
| Saline alteratives, | 186 | 94 | 50.8 |

Class II.—*Treated by Astringents*, 365 *Cases:*

| Treated by | Cases. | Deaths. | Per centage. |
|---|---|---|---|
| Sulphuric acid, | 488 | 235 | 48.1 |
| Other mineral acids, | 27 | 11 | 40.7 |
| Chalk, with or without opium, | 201 | 55 | 27.3 |
| Acetate of lead and opium, | 81 | 50 | 61.7 |
| Opium, | 36 | 11 | 30.5 |
| Iron and alum, | 13 | 6 | 46.1 |
| Gallic acid, etc., | 19 | 5 | 26.3 |

Class III.—*Treated by Stimulants*, 548 *Cases:*

| Treated by | Cases. | Deaths. | Per centage. |
|---|---|---|---|
| Ammonia, | 114 | 70 | 61.4 |
| Ether, | 154 | 65 | 42.1 |
| Alcohol, | 138 | 87 | 63. |
| Chloroform, | 31 | 15 | 48.3 |
| Other stimulants, | 111 | 50 | 45. |

Class IV.—*Treated by Evacuants*, 172 *Cases:*

| Treated by | Cases. | Deaths. | Per centage. |
|---|---|---|---|
| Castor oil, | 150 | 107 | 69.3 |
| Emetics, | 21 | 17 | 80.9 |

However carefully these data may have been collected, it will be wise not to draw positive conclusions from them, a piece of advice we need to offer to our younger readers only. Let them remember that the remark of Borrhaave, *Nullum ego cognosco remedium nisi quod tempestivo usu fiat tale*, is as applicable to cholera as to other diseases.—*The Stethoscope*, (*Va.*) *July.*

